# Comparative analysis of milk production, composition, and plasma metabolomics across various dairy goat breeds in Yunnan Province, China

**DOI:** 10.3389/fvets.2025.1552786

**Published:** 2025-06-12

**Authors:** Xun Xiang, Hua Chang, Samiullah Khan, Ibrar Muhammad Khan, Feiyan Zhu, Linfu Yang, Gang Duan, Jiming Li, Nourhan Nassar, Feiyan Dai, Huaming Mao

**Affiliations:** ^1^College of Animal Science and Technology in Yunnan Agricultural University, Kunming, China; ^2^College of Veterinary Medicine in Yunnan Agricultural University, Kunming, China; ^3^Guizhou Provincial Key Laboratory for Agricultural Pest Management of the Mountainous Region, Institute of Entomology, Guizhou University, Guiyang, China; ^4^School of Life Science, Anhui Agricultural University, Hefei, China; ^5^Animal Disease Prevention and Control Center of Shilin County, Shilin County Agricultural and Rural Bureau, Kunming, China

**Keywords:** milk yield, composition, plasma metabolomics, LC-MS technology, dairy goat

## Abstract

The present study involved crossing Alpine goats with Yunshang black bucks from Yunnan Province to produce Alpine-Yunshang dual-purpose F1 (AYF1) goats. The AYF1 goats were compared with Saanen and Toggenburg goats by evaluating milk yield, composition, and molecular markers in order to identify metabolites using liquid chromatography–mass spectrometry (LC–MS) technology. A total of 18 goats were selected, with 6 goats from each breed forming 3 groups. Daily milk yield was recorded, and milk composition was analyzed on days 60 and 120 of lactation. The results showed that on day 60, Saanen and AYF1 goats had significantly higher protein and fat contents than Toggenburg goats. On day 120, AYF1 goats had higher fat content, while Saanen goats had higher protein content compared to Toggenburg goats. There were no differences (*p* > 0.05) in milk yield among the breeds during the first, second, and fourth months; however, the Saanen breed yielded more milk during the third month compared to the other breeds (*p* < 0.05). Metabolomics analysis revealed a total of 1,108 distinct metabolites in the positive ion mode and 360 in the negative ion mode. The majority of these metabolites were found at higher levels in Saanen goats compared to other breeds. The relative abundance of 2-phenylacetamide, 5-aminolevulinic acid, and ureidopropionic acid was significantly higher in Saanen goats compared to AYF1 goats (*p* < 0.05). In the initial screening, 55 common and 17 useful differential metabolites were identified. A total of 17 metabolites, including 12 metabolic pathways and 7 functional classifications, were investigated using the KEGG platform. Metabolomics analysis showed that Saanen dairy goats produced higher levels of L-fucose, 4-acetamidobutanoic acid, L-tyrosine, and D-galactose, suggesting their involvement in milk production. In contrast, AYF1 goats had higher levels of sucrose, L-proline, ectoine, and biotin, suggesting their possible role in the metabolism of milk constituents. These findings offer insights into breed-specific milk metabolite profiles, highlighting the potential of AYF1 goats as a beneficial dual-purpose genotype for enhanced dairy and metabolic performance.

## Introduction

Small ruminants play a crucial role in production systems, particularly in developing regions of Asia, Africa, and Latin America (1). These animals are raised primarily by poor, small-scale producers and are regarded as valuable assets for smallholders, helping to generate income, secure food availability, and enhance resilience to climate change (2, 3). The rearing of these animals significantly contributes to household food security and promotes sustainable economic development. These animals play a crucial economic and ecological role in smallholder farming systems and agricultural practices (4).

Goat milk is increasingly recognized for its high nutritional content, ease of digestion, and better tolerance in individuals who are allergic to cow’s milk (5). It is considered a functional food, providing various health benefits due to its richness in medium-chain fatty acids, oligosaccharides, essential amino acids, and highly bioavailable minerals (6). On average, goat milk contains approximately 12.2% total solids, with nearly 3.8% fat, 3.5% protein, 4.1% lactose, and 0.8% ash (7). In addition, the smaller particle size of goat milk fat globules makes them easier to digest compared to those found in cow milk (8).

Metabolic profiling technologies have emerged as essential tools for investigating physiological traits, dietary effects, and animal health (9). This approach involves a systemic investigation of both endogenous and exogenous metabolites in biological fluids, utilizing modern analytical chemistry techniques such as liquid chromatography–mass spectrometry (LC-MS) (10, 11). By enabling the easy collection of biofluids and tissues, this method offers a comprehensive approach to the differentiation of mammalian milk based on the composition of small molecules (12, 13). Despite the extensive use of metabolomics technology, studies on goat milk are limited, which ultimately contrasts with the significance of goat milk in the global agricultural food economy (14, 15). However, identifying the molecular metabolites in goat milk can provide insights into various aspects of production and compositional characteristics of animals, providing opportunities for future advancements in the development of dairy goats (10).

Goats are predominantly reared for meat and dairy products, such as goat milk cake, which serves as a primary source of income for smallholder farmers in Yunnan province (16–18). A growing number of upland farmers in this region rely on livestock as a source of cash income, social security, a safeguard for future expenditures, and support for marriages and education. The majority of farmers reported that livestock farming serves as their main source of income in this region (16). In addition, the diverse geographical environment of this province makes it an ideal site for livestock production (19). Therefore, studying milk production and composition traits in different goat breeds can provide insights for genetic improvement and the development of nutritional strategies in dairy goat breeding.

The Yunshang black goat, a locally recognized meat breed in Yunnan, is known for its black coat, strong stress resistance, adaptability to rough forage, and moderate milk production (20). In this study, Alpine goats were crossed with Yunshang black bucks to produce Alpine-Yunshang dual-purpose F1 (AYF1) goats, and LC–MS-based metabolic profiling was employed to compare their milk composition with Saanen and Toggenburg goats for the identification of breed-specific biomarkers and metabolic pathways.

## Materials and methods

### Animal ethics statement and experimental design

In this study, we utilized Yunshang black goats, a recently developed and approved breed in China, primarily selected for meat production traits (20). Alpine goats (dairy type) were mated with Yunshang black bucks (meat type) to produce dual-purpose F1 (AYF1) goats. A total of 18 goats were selected from a larger population maintained at a commercial farm in Yunnan Province, China. The experimental groups included Saanen, Toggenburg, and Alpine-Yunshang dual-purpose F1 (AYF1) goats. From each breed, six goats of similar age (approximately 24.15 ± 0.7 months) and body condition were selected, ensuring uniformity in physiological status across the groups. All selected goats gave birth within the same period to maintain consistency in the lactation stage during sampling. Natural mating was practiced within each breed group using male goats of the same genetic background. All goats were housed in individual pens under similar environmental and management conditions, with ad libitum access to feed and clean water (Table 1) to meet the nutrient requirements. The experimental goats were milked twice daily, in the morning and evening.

**Table 1:** Ration ingredients and proximate composition of the experimental diets.

Ration ingredients, % dry matter (DM) basis	
Items (%)	
Alfalfa hay	10.64
Oat grass hay	9.81
Corn silage	17.83
Corn flour	19.68
Fresh alfalfa	5.16
Goat concentrate supplement	36.71
Limestone	0.21
Proximate composition and nutritive value, % DM	
Crude protein (CP)	14.27
Metabolizable energy, Mcal/kg DM	2.72
Ether extract (EE)	2.75
Ash	6.88
Nutrient detergent fiber (NDF)	30.90
Acid detergent fiber (ADF)	20.78
Ca	0.71
P	0.50

Premix provided per kg of diet: vitamin A 1,000 IU, vitamin D 200 IU, vitamin E 20 IU, iron 40 mg, copper 15 mg, iodine 2 mg, and manganese 40 mg.

### Milk yield and composition

The milk yield of all goat breeds that gave birth during the same period was recorded daily for 4 months. Manual milking was performed twice daily on the experimental animals. To investigate milk composition, milk samples were collected in the morning on days 60 and 120, with a 2-month interval. All these samples were stored at 4°C and then kept at 20°C in the laboratory until further analysis. Milk composition components, including fat, protein, lactose, total solids, solids-not-fat, and urea, were measured using a MilkoScan FT-120 (FOSS Electric A/S, Denmark).

### Plasma metabolism parameters

On day 120 of lactation, 5 mL of blood was collected from each animal into EDTA-coated tubes. Plasma was separated by centrifugation at 4,000 rpm for 15 min and stored at 4°C. A non-targeted LC-MS-based metabolomics approach was employed to analyze small molecule components in these samples.

### Metabolomics sample preparation

The samples were prepared according to the procedure previously described (21). First, the samples were thawed at 4°C and mixed thoroughly. To each sample, 400 μL of methanol was added, and these samples were then stored at −20°C for further processing. After 10 min of centrifugation at 12,000 rpm, the supernatant was transferred into a new 2-mL centrifuge tube. A solution of 2-chloro-l-phenylalanine (4 ppm) was prepared with 80% methanol–water to re-dissolve the sample, and the supernatant was filtered through a 0.22 μm membrane and transferred to a detection bottle for LC-MS detection analysis.

### Liquid chromatography conditions

Liquid chromatography was performed on an ACQUITY UPLC® HSS T3 column (150 × 2.1 mm, 1.8 μm; Waters, Milford, MA, United States) at 40°C using an ACQUITY UPLC System. The injection volume was 2 μL, with a flow rate of 0.25 mL/min. LC-ESI (±)-MS analysis utilized mobile phases of acetonitrile with 0.1% formic acid and water with 0.1% formic acid. Chromatographic separation followed a gradient program: 0–1 min, 2%; 1–9 min, 2–50%; 9–12 min, 50–98%; 12–13.5 min, 98%; 13.5–14 min, 98–2%; and 14–20 min, 2%. Acetonitrile and 5 mM ammonium formate were used for the LC-ESI (−)-MS analysis with the following gradient: 0–1 min, 2%; 1–9 min, 2–50%; 9–12 min, 50–98%; 12–13.5 min, 98%; 13.5–14 min, 98–2%; and 14–17 min, 2% (22).

### Mass spectrum conditions

Metabolite detection was performed using a Q Exactive™ mass spectrometer (Thermo Fisher Scientific, United States) equipped with an electrospray ionization (ESI) source. Data were acquired in the Full MS data-dependent MS/MS (ddMS^2^) mode. The sheath gas flow rate was set to 30 arbitrary units (arb), and the auxiliary gas flow rate was set to 10 arb. Spray voltages were +3.50 kV for the positive ion mode (ESI^+^) and −2.50 kV for the negative ion mode (ESI^−^). The capillary temperature was maintained at 325°C. The MS^1^ scan range was set between m/z 81 and 1,000 with a resolving power of 70,000 (FWHM), followed by 10 data-dependent MS^2^ scans per cycle with a resolving power of 17,500 (FWHM) (23).

### Quality control

To ensure analytical consistency, quality control (QC) samples were prepared by pooling equal volumes of extracts from all individual samples. Blank and QC samples were initially analyzed to evaluate system stability and reproducibility. During the run, all experimental samples were injected in a randomized order, with the QC samples interspersed at regular intervals after every three injections.

### Metabolite data processing and multivariate analysis

The raw data were converted to the mzXML format using the MSConvert function within the ProteoWizard software package. Subsequently, the data underwent feature detection, retention time adjustment, and alignment using XCMS (22). Metabolites were identified by comparing accurate mass (30 ppm) and MS/MS data to databases such as the Human Metabolome Database (HMDB) (24),^1^ MassBank (25), LIPID MAPS (26, 27), mzCloud, and KEGG (28). To correct any systematic bias, quality control-based robust LOESS signal correction (QC-RLSC) (29) was applied during data normalization. For accurate metabolite identification post-normalization, only ion peaks with relative standard deviations (RSDs) below 30% in quality control (QC) were retained.

Analysis and modeling using multivariate data were carried out utilizing Ropls software (30). After data normalization, statistical models incorporating principal component analysis (PCA), orthogonal partial least squares discriminant analysis (OPLS-DA), and partial least squares discriminant analysis (PLS-DA) were developed. The metabolic profiles were represented in score plots where each point denoted a unique sample. Loading plots and S-plots were utilized to highlight key metabolites influencing the clustering patterns. To prevent overfitting, the models underwent thorough evaluation via permutation testing. The assessment of model performance included the examination of cumulative R2X and R2Y values for descriptive purposes [ideal R2X (cum) = 1], as well as cumulative Q2 values alongside permutation testing for predictive capabilities [ideal Q2 (cum) = 1]. To ensure accuracy, the non-permuted model should exhibit higher Q2 and R2 values at the Y-axis intercept compared to the permuted model. Through variable importance in projection (VIP) analysis, significant metabolites were identified using OPLS-DA, with noteworthy variables defined by *p*-values < 0.05 and *VIP* values > 1.

### Pathway analysis

As indicated (31), MetaboAnalyst evaluated several metabolites for pathways using robust pathway enrichment analysis and pathway topology analysis. The metabolites found in the metabolomics analysis were mapped using the KEGG pathway for the biological interpretation of higher systemic functions. The metabolic pathways and related metabolites were visualized using the KEGG Mapper program.

### Statistical analysis

Milk yield and composition data were analyzed using one-way analysis of variance (ANOVA) in SPSS Statistics 18.0 to assess differences between the goat breeds. When significant differences were detected (*p* < 0.05), Tukey’s Honest Significant Difference (HSD) *post-hoc* test was employed to identify specific group differences. The results are presented as means ± standard deviations, with statistically distinct groups denoted by different lowercase letters.

For the metabolomics data, multivariate statistical analyses, including principal component analysis (PCA), partial least squares discriminant analysis (PLS-DA), and orthogonal PLS-DA (OPLS-DA), were performed using the R package ropls. Significant metabolites were identified based on variable importance in projection (VIP) scores greater than 1 and *p*-values less than 0.05. Pathway enrichment and topology analyses were carried out using MetaboAnalyst, with metabolite mapping conducted via the KEGG database.

## Results

### Milk composition and yield in the dairy goats

**Figure 1:**
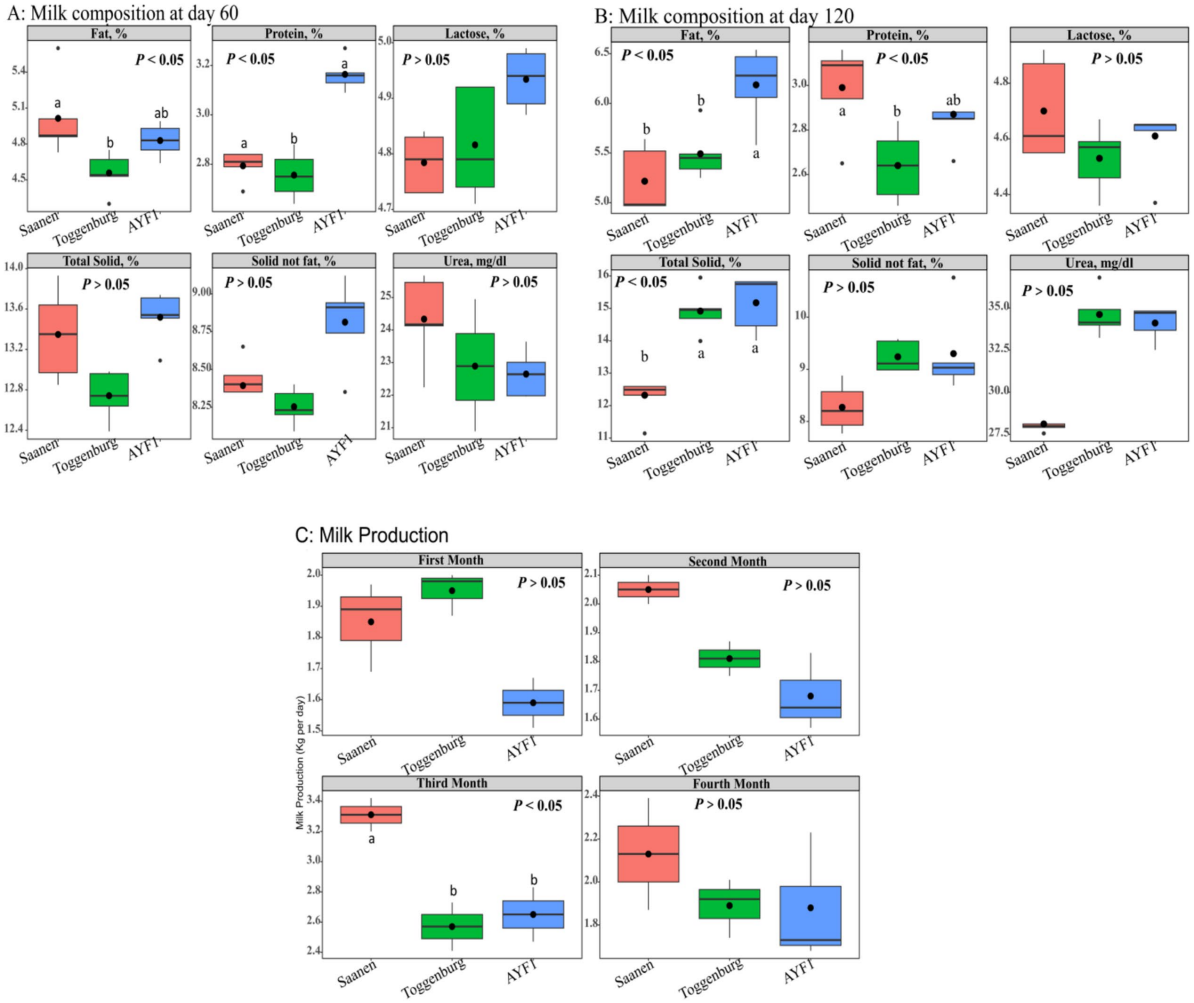
**(A)** Milk composition of the different goat breeds on day 60 of lactation, **(B)** milk composition of the different goat breeds on day 120 of lactation, and **(C)** milk yield of the different goat breeds during the first 4 months of lactation.

Milk fat, protein, lactose, total solids, non-fat solids, and urea concentrations in the experimental groups were analyzed on days 60 (Figure 1A) and 120 of lactation (Figure 1B). On day 60 of lactation, the AYF1 and Saanen goats (2.79 and 3.16%) had significantly higher milk protein contents (*p* < 0.05) compared to the Toggenburg (2.75%) goats. Similarly, the Saanen goats had significantly higher fat content than the Toggenburg goats (*p* < 0.05). There were no significant differences (*p* > 0.05) observed among the groups for the other milk composition traits. On day 120 of lactation, the AYF1 goats exhibited (*p* > 0.05) higher milk fat content compared to the Saanen and Toggenburg goats, while the Saanen goats had higher protein content (*p* > 0.05) than the Toggenburg goats.

No differences were observed among the different groups for milk yield during the first, second, and fourth months of lactation. However, during the third month, the milk yield of the Saanen goats was found significantly (*p* < 0.05) higher than that of both Toggenburg and AYF1 goats (Figure 1C).

### Metabolite data quality control and quality assurance

**Figure 2:**
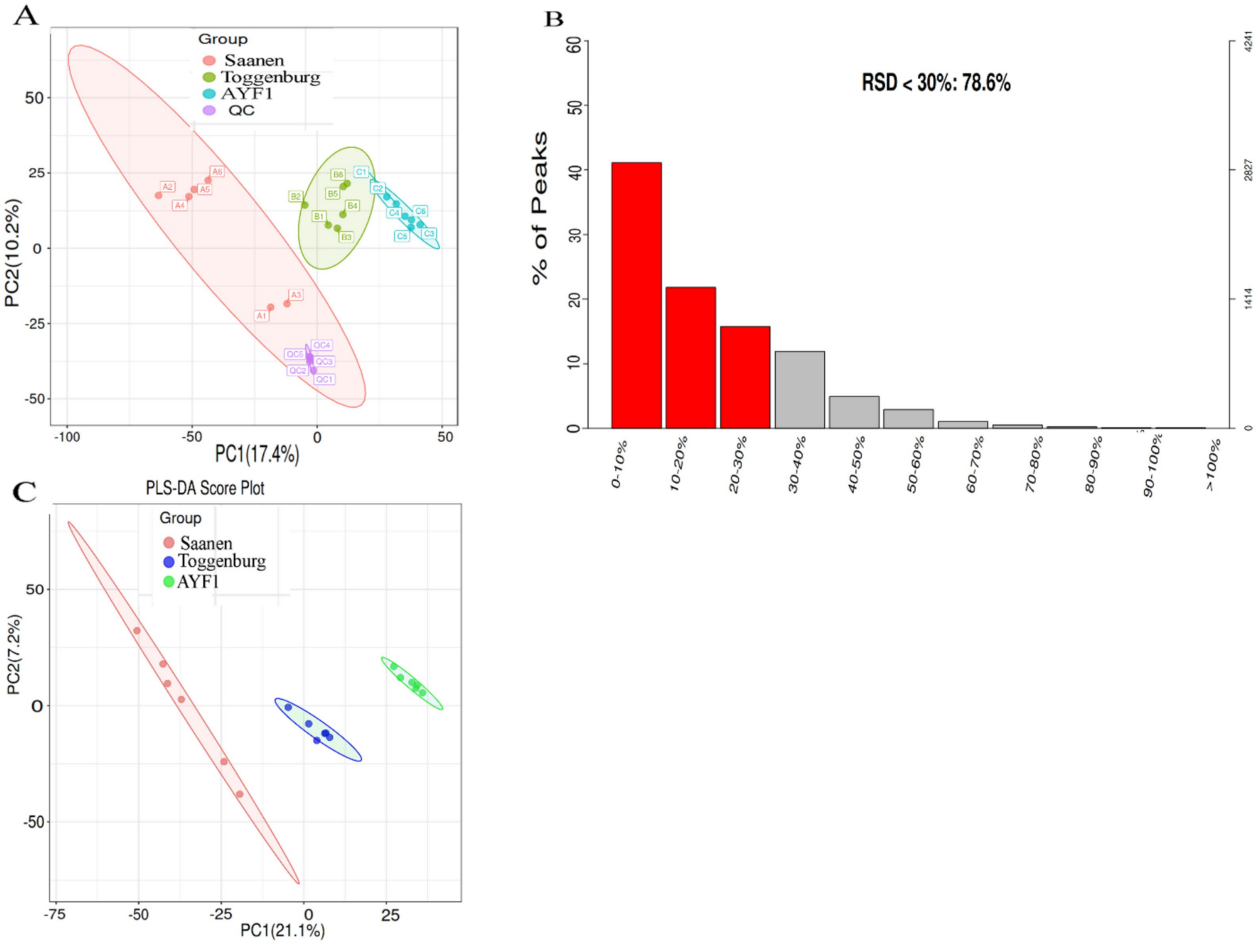
**(A)** PCA score plot of the metabolite data for the QC sample. The red point represents the QC sample, while the colored points represent the samples from the three different breeds. QC stands for quality control; PCA stands for *principal component analysis.* The axes that represent the variations are “principal Components,” with PC1 accounting for the greatest variation in the data and PC2 accounting for the second greatest variation. **(B)** The signature peak with an RSD < 30% in the metabolome of this trial dataset accounted for 83.5%. The quality assurance (QA) results show the distribution of RSD (relative standard deviation %) in the metabolomics data. The left ordinate represents the proportion, the right ordinate represents the specific number, and the abscissa represents the range of the RSD values. **(C)** PLS-DA scoring chart. The first principal component interpretation is represented by the abscissa, and the second principal component interpretation is represented by the ordinate direction. The experimental samples are represented by points, with different colors indicating the respective groups. The samples between the groups are more dispersed, indicating that the results are more reliable when the samples within each group are more aggregated.

Quality control (QC) is necessary in metabolomics research based on mass spectrometry to obtain reliable, high-quality metabolomics data. In this study, mixed QC samples were used for QC in LC–MS detection. The non-targeted metabolite detection approach offered great stability and better data quality, as shown by the small differences between the QC samples and aggregated data. The PCA analysis chart’s dense distribution of the QC samples demonstrated the validity of the data (Figure 2A). The proportion of typical peaks with a relative standard deviation (RSD) of 30% in the QC samples reached 83.5%, as shown by the quality assurance analysis of the metabolic group data (Figure 2B), demonstrating the reliability of the data. The metabolomics data were clustered using PLS-DA, a supervised pattern recognition technique. The data from each experimental group were evenly distributed and concentrated, demonstrating the validity of the metabolomics data, of which PC1 represented 21.1% and PC2 represented 7.2% (Figure 2C).

### Variance analysis

After confirming the accurate molecular weight based on MS/MS fragments, we identified and annotated metabolites using standard databases, such as the Human Metabolome Database, MassBank, LIPID MAPS, and mzCloud. Differential metabolites were identified from the major substance list. The *p*-value and *VIP* threshold in the statistical test were screened, and the results of the screening are presented in Table 2 (Supplementary Figure S1). A total of 38 differential metabolites were identified between the Saanen and Toggenburg goats, including 24 upregulated and 14 downregulated metabolites. Between the Saanen and AYF1 goats, 68 metabolites were significantly altered, with 47 upregulated and 21 downregulated. The comparison between the Toggenburg and AYF1 goats showed 34 differential metabolites (15 upregulated and 19 downregulated). In total, 55 unique metabolites were found, which were differentially expressed across all three breeds.

**Table 2:** Statistical summary of the differential metabolites among the different goat breeds.

Comparison	Upregulated	Downregulated	Total
Saanen vs. Toggenburg	24	14	38
Saanen vs. AYF1	47	21	68
Toggenburgy vs. AYF1	15	19	34
Saanen vs. Toggenburg vs. AYF1	/	/	55

**Figure 3:**
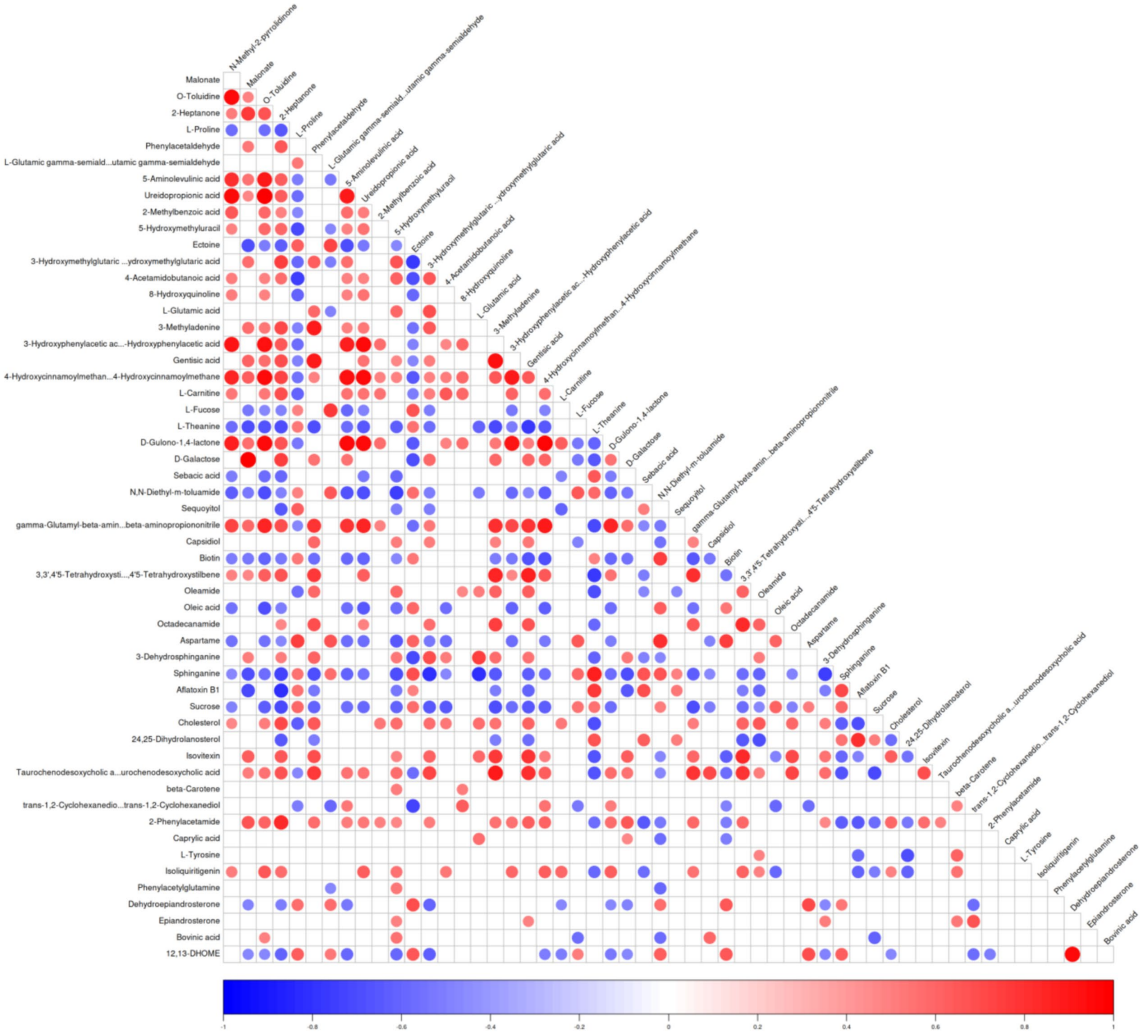
Differential metabolite-associated thermogram. The ordinate and oblique ordinate represent the names of the differential metabolites, and the color represents the correlation, with red representing positive correlations, blue representing negative correlations, and the intensity of the color reflecting the strength of the correlation.

A correlation heatmap was constructed to visualize the relationships among the identified differential metabolites across all experimental goat groups. The heatmap shows the distribution of the 55 unique metabolites across the three experimental groups (Figure 3; Supplementary Figure S2). Each square represents the Pearson correlation coefficient between a pair of metabolites, with red indicating positive correlations and blue indicating negative correlations. The intensity of the color and the size of the circle correspond to the strength of the correlation, ranging from −1 (strong negative) to +1 (strong positive). The majority of the differential metabolites in the Saanen goats were substantially higher compared to the Toggenburg and AYF1 goats. Of the 55 shared differential metabolites across the experimental groups, 17 useful differential metabolites were screened by eliminating those with limited biological relevance. These selected metabolites were further subjected to one-way ANOVA for comparative analysis (Table 3). Among the 17 screened differential metabolites, the majority were more abundant in the Saanen goats compared to the Toggenburg and AYF1 goats. Among these metabolites, D-galactose, cholesterol, 5-Aminolevulinic acid, trans-1, 2-cyclohexanediol, and L-tyrosine were more abundant in the Saanen goats compared to the AYF1 goats, while sucrose, L-proline, ectoine, and biotin were significantly higher in the AYF1 goats than in the other breeds (*p* < 0.05). Pathway analysis and functional classification of the selected metabolites were performed on the KEGG platform, with 12 metabolic pathways and seven functional classifications across the 17 metabolites. These included the following: two benzene and alternative derivatives, three carboxylic acids and derivatives, two organic oxygen compounds, two steroids and steroid derivatives, one fatty acyl and its derivatives, and one biotin and its derivative (Tables 4, 5).

**Table 3:** Results of the single-factor analysis of variance for the different metabolites across the different groups.

Metabolites	Saanen breed	Toggenburg breed	AYF1 breed
L-fucose	30232419 ± 1271835.78^a^	25318266.56 ± 2892473.88^b^	24816769.20 ± 3403230.84^b^
Malonate	201440256.35 ± 54129261.47^a^	142312546.78 ± 30430103.11^a^	114857962.97 ± 38126235.28^b^
4-Acetamidobutanoic acid	29059153.19 ± 4931502.57^a^	23704911.88 ± 9271683.42^b^	15177384.48 ± 4354388.38^b^
Ureidopropionic acid	23007959.28 ± 3246752.47^a^	20595990.91 ± 3810303.1^a^	15827511.7 ± 2763194.73^b^
Gentisic acid	3004911550.67 ± 616284251.54^a^	2,226,911,484 ± 264457067.08^ab^	2081641593.17 ± 112665217.84^b^
D-galactose	233895971.88 ± 63708649.17^a^	172831094.27 ± 29816363.1^b^	139264033.53 ± 46295539.04^c^
Sucrose	1365029.71 ± 854019.68^c^	3760539.71 ± 841319.2^b^	4858725.03 ± 1819232.3^a^
Cholesterol	12660516.65 ± 4616415.09^a^	7055410.95 ± 2786037.83^b^	4922879.17 ± 2831400.98^b^
L-proline	14121713.65 ± 8877933.06^c^	32516403.74 ± 26149426.31^b^	63294201.05 ± 39648346.13^a^
5-Aminolevulinic acid	426163498.93 ± 50827962.48^a^	415842577.83 ± 61996645.14^a^	288456833.07 ± 67864614.31^b^
Ectoine	43811115.69 ± 13777253.06^c^	101142375.75 ± 61998650.33^b^	241052988.25 ± 104465671.53^a^
Biotin	5060934.01 ± 2311568.4^c^	5525142.74 ± 1938525.56^b^	9538274.67 ± 1962357.79^a^
2-Phenylacetamide	41922489.84 ± 2270294.52^a^	30961675.06 ± 6734126.32^a^	29028344.78 ± 5389058.17^b^
trans-1,2-Cyclohexanediol	7496221.85 ± 366173.4^a^	7360746.26 ± 317051.41^a^	6938964.89 ± 280565.16^b^
Caprylic acid	146671371.6 ± 71816689.81^a^	169,554,169 ± 181427578.26^a^	29481774.66 ± 17430931.24^b^
Dehydroepiandrosterone	4521055.26 ± 226486.31^b^	4288575.1 ± 324597.41^b^	5684838.89 ± 865282.91^a^
L-tyrosine	73079471.29 ± 21369170.07^a^	40305951.31 ± 6111608.91^b^	51693101.62 ± 9385396.69^b^

^a,b,c^Different superscripts within the rows indicate levels of significance at a *p*-value < 0.05 (Tukey HSD test).

**Table 4:** KEGG pathways and functions of the 17 differential metabolites in the different dairy goats.

Name	KEGG pathway	Functional role
5-Aminolevulinic acid	ABC transporters	Carboxylic acids and derivatives
4-Acetamidobutanoic acid	Arginine and proline metabolism	Carboxylic acids and derivatives
Malonate	Beta-Alanine metabolism	—
Ureidopropionic acid	Beta-Alanine metabolism	Organic carbonic acids and derivatives
Caprylic acid	Biosynthesis of alkaloids derived from terpenoid and polyketide	Fatty Acyls
L-proline	Carbapenem biosynthesis	Carboxylic acids and derivatives
Cholesterol	Cholesterol metabolism, Steroid degradation	Steroids and steroid derivatives
Ectoine	Microbial metabolism in diverse environments	—
L-fucose	Microbial metabolism in diverse environments	Organooxygen compounds
Trans-1,2-Cyclo-hexanediol	Microbial metabolism in diverse environments	—
2-Phenylacetamide	Microbial metabolism in diverse environments	Benzene and substituted derivatives
D-galactose	Mineral absorption	—
Dehydroepiandrosterone	Ovarian steroidogenesis	Steroids and steroid derivatives
L-tyrosine	Protein digestion and absorption	Carboxylic acids and derivatives
Sucrose	Taste transduction, Carbohydrate digestion and absorption	Organooxygen compounds
Gentisic acid	Tyrosine metabolism	Benzene and substituted derivatives
Biotin	Vitamin digestion and absorption, ABC transporters	Biotin and derivatives

**Table 5:** Distribution of the main differential metabolites in the KEGG pathway across the different dairy goats.

Pathway name	Total	Hits	*p*-value	Impact	Compound name
Phenylalanine metabolism	60	5	0.0002	0.0833	L-tyrosine; Phenylacetaldehyde; 2-Phenylacetamide; Phenylacetylglutamine; 3-Hydroxyphenylacetic acid
Styrene degradation	24	3	0.0012	0.125	Phenylacetaldehyde; 2-Phenylacetamide; 3-Hydroxyphenylacetic acid
Carbapenem biosynthesis	32	3	0.0027	0.0938	L-Glutamic acid; L-proline; L-Glutamic gamma-semialdehyde
Cholesterol metabolism	10	2	0.0033	0.2	Cholesterol; Taurochenodesoxycholic acid
Vitamin digestion and absorption	39	3	0.0048	0.0769	Biotin; Cholesterol; beta-Carotene
Arginine and proline metabolism	78	4	0.0049	0.0513	L-Glutamic acid; L-proline; L-Glutamic gamma-semialdehyde; 4-acetamidobutanoic acid
ABC transporters	137	5	0.0071	0.0365	L-Glutamic acid; Sucrose; Biotin; L-proline; 5-Aminolevulinic acid
Fatty acid biosynthesis	58	3	0.0145	0.0517	Malonate; Oleic acid; Caprylic acid
Carbohydrate digestion and absorption	27	2	0.0236	0.0741	Sucrose; D-galactose
Mineral absorption	29	2	0.027	0.069	D-galactose; L-proline
Tyrosine metabolism	78	3	0.0317	0.0385	L-tyrosine; Gentisic acid; 3-Hydroxyphenylacetic acid
Beta-Alanine metabolism	32	2	0.0325	0.0625	Malonate; Ureidopropionic acid

Total, the total number of metabolites in the target metabolic pathway; Hits, the total number of differential metabolites in the target metabolic pathway; *p-*value, the *p*-value of the hypergeometric distribution test—the smaller the *p*-value, the more significant the effect of the detected differential metabolites on the pathway; Impact, the greater the impact value of the metabolic pathway, the greater the impact of the detected differential metabolites on the target pathway; compound name, the name of the differential metabolites detected in the target metabolic pathway.

## Discussion

The results comparing the milk composition of the different dairy goat breeds showed that milk protein differed between the Toggenburg and AYF1 goats. However, the milk contents of the three dairy goats were comparable for other indices under identical feeding conditions. The Saanen and Toggenburg goats had similar protein, lactose, and total solids contents, but their fat content was higher than previously recorded for the same breeds (32). Variation in the composition of milk has been reported across different breeds (32–35). Previous research indicated that the Nubian breed exhibited higher levels of milk fat (4.37%), total protein (3.87%), and total solids (13.5%), whereas the Alpine breed displayed lower values for milk fat (2.7%), total protein (2.53%), and total solids (10.1%) (36). Similarly, variations in milk yield and composition have been reported among Arsi-Bale, Somali, Toggenburg-Arsi-Bale cross, and Boer goats at different lactation stages. Arsi-Bale goats exhibited a protein content of 4.8%, which was significantly higher than that of the other breeds. Milk from the crossbreed had lower total solids (13.88%) and fat (3.65%) compared to Arsi-Bale, Boer, and Somali goats, which had 16.27, 15.44, and 14.48% total solids and 5.15%, 4.70%, and 4.90% fat, respectively (33). A notable variation in milk composition was observed, with the Anglo-Nubian breed exhibiting higher levels of fat (4.25%), protein (3.4%), and total solids (12.5%). This was followed by the Saanen breed, raised in Southeastern Brazil, and the Alpine breed, which averaged 3.7% fat, 2.95% protein, and 11.8% total solids (34). Several factors have been reported to influence milk components, such as breeds (37), seasonality across different regions of the world (38), production systems (39), climate, nutritional quality of food, and other management factors (37).

In this study, pathway analysis and functional classification of the selected metabolites were carried out using the KEGG platform. A total of 17 metabolites were analyzed, and these included two benzene and alternative derivatives, three carboxylic acids and derivatives, two organic oxygen compounds, two steroids and steroid derivatives, one fatty acyl and its derivative, and one biotin and its derivative. It has been reported that L-proline, 5-Aminolevulinic acid, ectoine, and biotin play vital functions in animals (40). The AYF1 goats exhibited notably higher levels of L-proline compared to the Saanen and Toggenburg goats, which may be associated with enhanced disease resistance (41). The Saanen goats exhibited notably higher concentrations of gentisic acid and D-galactose compared to the other groups. Gentisic acid possesses cardioprotective effects; for example, in individuals with compromised lung structure, it can delay the progression from cardiac hypertrophy to heart failure. It achieves this by suppressing the renin–angiotensin–aldosterone system (RAAS), thereby preventing cardiac dysfunction and fibrosis. Due to its low toxicity, gentisic acid may hold potential as a preventive or therapeutic agent against heart failure (42).

In the comparison of the differential metabolites among the three dairy goat breeds, biotin levels in the AYF1 dairy goats were significantly higher than in the other two breeds. Recent studies have documented the involvement of biotin in functions beyond its usual catalytic functions. The effects of pharmaceutical biotin on glucose and lipid metabolism, hypertension, reproduction, development, and immunity have been established in multiple studies (40). It can be speculated that the stress resistance of the AYF1 goats was significantly better than that of the other two breeds. Caprylic acid levels were observed to be higher in the Toggenburg goats compared to the other groups. Goat milk is known to be rich in medium-chain fatty acids, especially caprylic acid (C8:0) and capric acid (C10:0). Caprylic acid is metabolized quickly for energy and has antimicrobial properties (7).

In the present study, 55 common differential metabolites were detected across the three experimental groups. Among these differential metabolites, 17 metabolites were selected as informative through a screening process. These metabolites were further analyzed using the KEGG platform for pathway mapping and functional categorization, resulting in the identification of 12 metabolic pathways and seven functional classifications associated with the selected metabolites. A study was conducted to assess the impact of specific grazing patterns and their corresponding nutritional effects on goat milk (5). Using gas chromatography–mass spectrometry (GC-MS) techniques, the authors identified and quantified 25 distinct milk metabolites in milk. Our study showed that the Saanen goats had significantly elevated levels of L-fucose, 4-acetamidobutanoic acid, D-galactose, and L-tyrosine compared to the Toggenburg and AYF1 goats. This suggests that these four metabolites may contribute to milk production. In addition, sucrose, L-proline, ectoine, and biotin were more pronounced in the AYF1 dairy goats compared to the other two groups. This suggests that these four metabolites may be involved in the control and metabolism of milk constituents.

### Clinical significance of the findings

The main clinical findings of the current investigation indicate that distinct metabolites impact the metabolism of various breeds when subjected to the same environmental conditions. This influence can enhance their ability to modulate milk output and composition. Our study investigated the potential roles of sucrose, L-proline, ectoine, and biotin in the regulation and metabolism of milk constituents, as well as the performance and composition of milk in dairy goats. The relative abundance of L-fucose, 4-acetamidobutanoic acid, D-galactose, and L-tyrosine was shown to be higher in the Saanen dairy goats compared to the other two groups. This observation suggests a potential association between these four metabolites and milk production. Further investigation is required to annotate the LC-MS signals corresponding to these metabolites. This can be achieved by increasing the number of samples collected monthly. This study employed LC-MS technology to establish molecular markers for subsequent variety screening and to identify potential metabolites in the plasma of the dairy goats subjected to identical settings and conditions.

Examining these metabolic pathways may provide insights into dairy products’ *in vivo* metabolism. Gaining a comprehensive understanding of milk metabolites derived from various dairy breeds could prove to be an excellent resource in the assessment of milk characteristics. This could also improve formula quality and facilitate the development of formulas that closely resemble human milk. The current study has the potential to serve as a reference point for the advancement and evaluation of commercial baby formulas and functional dairy products. Furthermore, these findings will be utilized as the foundation for our subsequent inquiry into the serum metabolomics of milk from these specific breeds. Milk metabolites are associated with specific dairy animal species and impact the nutritional composition of infant formulas. The objective of this study was to investigate the variations in metabolites present in the milk of different dairy goat breeds using LC/MS metabolomics. The aim was to gain a comprehensive understanding of the metabolomes from multiple milk sources. In addition, it was to identify unique metabolic pathways across goat breeds. This work aimed to elucidate the different functional constituents present in milk. This will establish a basis for the development of a powdered formula for newborns that closely mimics human milk, promoting optimal infant well-being. The data enhanced our understanding of the potential metabolic pathways that may explain the differences in milk performance observed among the Saanen, Toggenburg, and AYF1 goats. The findings will offer novel enhancement strategies, along with new possibilities for growth within the dairy industry.

## Conclusion

This study showed that the Saanen breed produced more milk than the Toggenburg and AYF1 goats. Through metabolomic differential analysis, four metabolites, including L-fucose, 4-acetamidobutanoic acid, D-galactose, and L-tyrosine, were identified as potentially linked to dairy goat productivity. Similarly, sucrose, L-proline, ectoine, and biotin may be associated with the regulation of milk composition. These results suggest that the identified metabolites could play key roles in enhancing and regulating animal milk production and constituents. Additional studies are warranted to confirm these initial findings and fully elucidate the roles of these metabolites. Further research with larger sample sizes for each group and expanded breed comparisons is recommended to validate these findings and better assess breed-specific performance traits.

## Data Availability

The original contributions presented in the study are publicly available. This data can be found here Metabolights, accession ID is MTBLS12574, https://www.ebi.ac.uk/metabolights/reviewer27ef7c25-8f08-416a-a2c4-d2fb8bebb25a.

## Footnotes

^1^ http://www.hmdb.ca
