# Supplementary material for: Comparative analysis of milk production, composition, and plasma metabolomics across various dairy goat breeds in Yunnan Province, China
Source: Front Vet Sci. 2025 Jun 12;12:1552786. doi: 10.3389/fvets.2025.1552786 (PMC12199267; doi:10.3389/fvets.2025.1552786)
Supplement: Supplementary file 1 [file Data_Sheet_1.docx]

**Supplementary Figures**

**Figure S1: Differential metabolites statistical histogram**


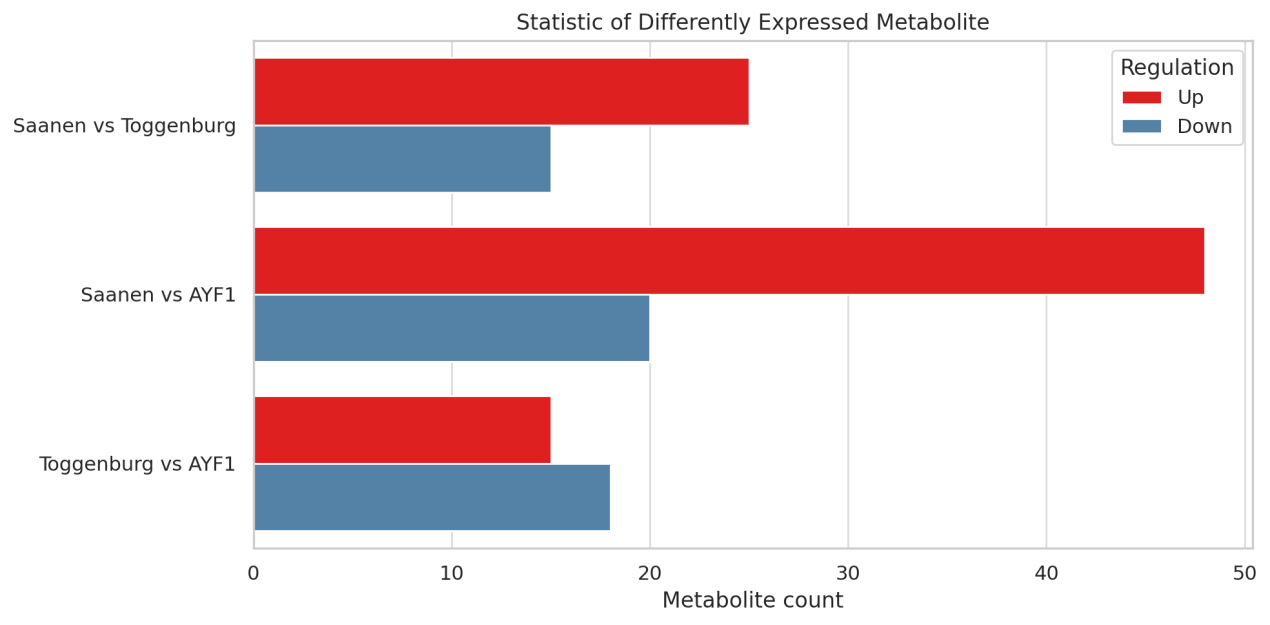


Differential metabolites statistical histogram in each group. X axis represents the number of differential metabolites, and Y axis represents the grouping comparison condition.

**Figure S2: Venn Diagram of differential metabolites in each group**


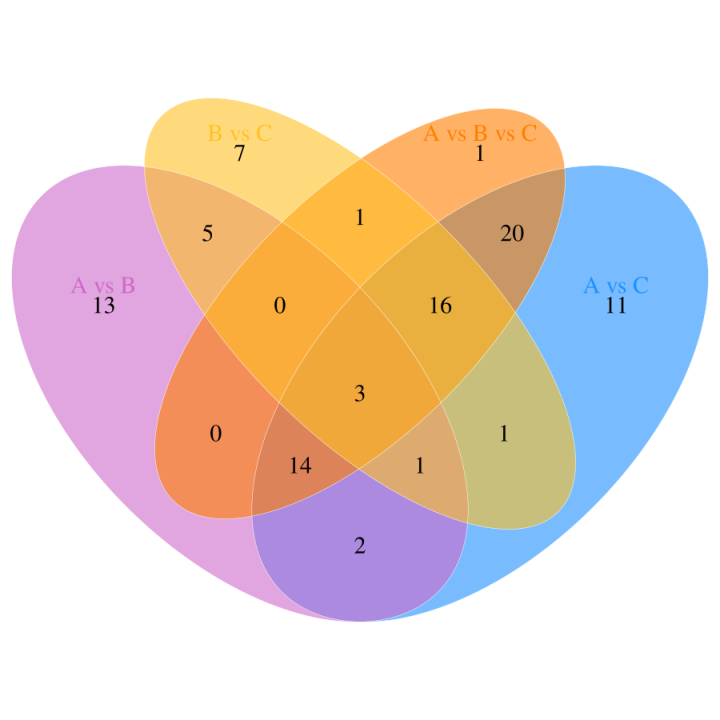


Different color regions represent differential metabolites of different groups, and overlapping regions are common differential metabolites of multiple groups. A, B, C represent Saanen, Toggenburg and AYF1, respectively.
